# Editorial: Molecular Biology of Archaea - 2022

**DOI:** 10.3389/fmicb.2024.1393932

**Published:** 2024-04-09

**Authors:** Marleen van Wolferen, Solenne Ithurbide, Michel Geovanni Santiago-Martínez, Arthur Charles-Orszag

**Affiliations:** ^1^Molecular Biology of Archaea, Institute of Biology II - Microbiology, University of Freiburg, Freiburg, Germany; ^2^Département de Microbiologie, Infectiologie et Immunologie, Université de Montréal, Montréal, QC, Canada; ^3^The Microbial Ecophysiology Laboratory, Department of Molecular and Cell Biology, University of Connecticut, Storrs, CT, United States; ^4^Department of Cellular and Molecular Pharmacology, Howard Hughes Medical Institute, University of California, San Francisco, San Francisco, CA, United States

**Keywords:** archaea, archaeal biology, genetics, omics, molecular biology, microscopy, extremophile, evolution

## Introduction

Since their identification and reclassification four decades ago, the study of microorganisms from the Domain Archaea has proven to be a continuous source of exciting discoveries, contributing to the characterization of their unique molecular mechanisms, metabolisms, ecophysiology, phylogeny, and cell biology (Spang et al., 2017; Lyu et al., 2018; Baker et al., 2020; van Wolferen et al., 2022). These findings have revealed the impact that archaea play in nutrient cycles, biotechnology advancements, and One-Health microbiomes (Pfeifer et al., 2021; Hoegenauer et al., 2022). In addition, the advancements in archaeal biology have highlighted the key position that archaea occupy in the evolution and diversification of the Tree of Life (Spang et al., 2017; Baker et al., 2020). The study of archaea has thereby brought us closer to elucidating the origin and early forms of microbial life, while also gaining insights into the limits of life detection.

Despite these important findings and the larger audience that archaea have consequently gained, many aspects of their biology remain unexplored. However, recent and ongoing developments in the field are addressing the technical limitations related to the isolation and cultivation. These advancements are allowing archaeal researchers to tackle open and upcoming questions. This promises exciting new discoveries in the near future that will continue to build on our understanding of the biology of archaea and allow us to uncover their unique features.

We are a team of early career scientists coming from different laboratories around the world. We are involved in the initiatives Archaea Power Hour as well as Archaea.bio, both aiming at promoting the diversity of researchers, supporting the exchange of ideas, and fostering collaborations in the field of archaeal biology. In collaboration with the organizers, Jörg Soppa, Sonja-Verena Albers, and Anita Marchfelder of the EMBO workshop for Molecular Biology of Archaea (August 1–4, 2022, Frankfurt, Germany), we have curated a collection of publications highlighting the current research trends in the Molecular Biology of Archaea and related topics. Our aim was to showcase the latest research findings and to encourage discussion about the advances, remaining challenges and future directions for researchers studying archaea.

With this goal in mind, we invited all participants of the workshop, as well as other archaeal researchers around the world, to submit their manuscripts on the molecular biology of archaea. We are pleased to have accepted 18 manuscripts that collectively contribute to our understanding of the biology of archaea. In this editorial, we provide a brief overview of these manuscripts, highlighting their key contributions to the field.

## Advances on Molecular Biology of Archaea

Organisms from the domain of Archaea were once thought to thrive exclusively in extreme environments, such as hot springs, hydrothermal vents, or salt flats. They however turned out to be ubiquitous and can actually be found in very diverse environments, from oceans to soils and even the host-associated microbiomes of plants, animals and the human gut. Archaea exhibit remarkable genetic diversity and metabolic versatility, exceeding that of bacteria. They thereby represent an immense -yet largely untapped- source of new microbial biology with potential applications in various fields. Evolutionarily, it is now commonly accepted that the eukaryotic lineage roots form within the archaea. From an ecological standpoint, archaea play crucial roles in global biogeochemical cycles and climate regulation. They are the main producers of Earth's methane, and their biomass (in gigatons of carbon) is thought to rival or even surpass that of animals, making them key players in global biogeochemical cycles and the Earth's climate. Yet, compared to bacteria and eukaryotes, their significance is often overlooked.

Since 2008, the international conference Molecular Biology of Archaea (MBoA) has been instrumental in advancing the field of archaeal research. By providing a platform for the exchange of new ideas, spurring scientific collaborations and, most importantly, welcoming new researchers to the field of archaea, the MBoA has significantly contributed to the rapidly evolving field of archaeal biology. This Research Topic showcases a selection of discoveries presented at the MBoA conference held in August 2022 in Frankfurt, Germany as well as contributions from researchers around the world. Covering a wide variety of topics across different biological scales, from environmental studies to controlled laboratory experiments, in both model and non-model organisms, the research showcased here illustrates the breadth typical of research endeavors in the biology of archaea.

In the past decade, advances in metagenomics and genomics have significantly enhanced our knowledge of archaeal biology. These techniques have enabled the identification of numerous new species and lineages without the need for traditional culture-based methods. This has had widespread implications for our understanding of archaeal phylogeny, ecology, physiology, and genetic diversity. The central and multifaceted role of genomics and other -omics approaches in archaeal research is well illustrated in this Research Topic. It includes the complete genome assembly of *Methanococcus aeolicus* PL15/Hp, relevant to biotechnology (Fomenkov et al.), the characterization of two novel extremely halophilic symbiotic nanohaloarchaea (Reva et al.), and the drawing up of an exhaustive taxonomy of methanogens associated with terrestrial arthropods intestinal tract (Protasov et al.).

Historically, the study of archaea is almost indiscernible from the study of microorganisms living in extreme environments (extremophiles). Ever since the discovery of the first hyperthermophiles in the early 1970s, various extremophilic archaeal species have successfully been cultivated. These organisms allowed the characterization of biological processes and molecular adaptations to extreme environments, such as high salt, high pressure, extreme temperatures and pH levels, oxygen-free environments, and others. More recently, the development of genetic tools in extremophilic archaea has revolutionized our ability to address functional questions in these organisms. This boom is reflected in a series of articles that dig into the physiology of thermophilic archaea.

Several of these studies cover transcriptional regulation and response, including: the regulation of archaellation in *Pyrococcus furiosus* by the highly conserved transcriptional regulator EarA (Stöckl et al.); the transcriptional regulation of denitrification in *Haloferax mediterranei* (Miralles-Robledillo et al.); the transcriptional response to the deletion of Nudix hydrolases in *Sulfolobus acidocaldarius* (Breuer et al.); and lipid modifications and differential expression in response to cold stress in *Saccharolobus islandicus* (Chiu et al.) and the role of zinc finger μ-protein HVO_0758 in *Haloferax volcanii* (Üresin et al.). Other studies focused on: tRNA modification in Archaeoglobi (Pichard-Kostuch et al.); metabolism of amino acids in *Thermococcus kodakarensis* (Su et al.); high frequency chromosomal gene transfer induced by an integrated conjugative plasmid in *Sulfolobus islandicus* (Sanchez-Nieves et al.). Notably, certain archaeal species, despite being unamenable to genetic manipulations, serve as key models to characterize enzymes involved in highly conserved processes such as DNA methylation in the extremophile *Picrophilus torridus* (Gulati et al.) and methanogenesis in *Methanothermococcus thermolithotrophicus* (Lemaire et al.).

The field of archaeal biology is now in a phase of rapid growth, with continuous development of new genetic methods, even in model species where genetic tools are already available. For example, an alternative method for gene deletion based on the CRIPSR-Cas system has now been introduced in the model *Sulfolobus acidocaldarius* (Bost et al.). In halophiles, a whole new set of xylose-inducible promoters has been introduced, providing valuable tools for research model haloarchaea (Rados et al.). Additionally, an improved system for in-frame gene deletion has been developed for the model species *Halorubrum lacusprofundi* (Gebhard et al.). These advancements represent significant progress in expanding the genetic toolkit available for studying archaeal biology.

Exploring the unique cell biology of archaea presents a vibrant and exciting new avenue of research, albeit with many challenges. For example, the study of archaeal cell shape maintenance is particularly complex due to pleomorphic nature of some haloarchaeal species. This complexity requires a rigorous survey of cell shapes changes that may solely arise from the experimental conditions, as shown in *Haloferax volcanii* (Patro et al.). Furthermore, the specific membrane lipid composition, growth temperature and pH characteristics typical of archaeal thermophiles, such as *Sulfolobus*, have posed significant challenges to imaging techniques. To address this, a vast repertoire of membrane and DNA dyes, commonly used to image bacteria and eukaryotes was tested in *Sulfolobus acidocaldarius*. This study serves as a crucial reference for future imaging studies in archaea, providing valuable insights into the selection of appropriate imaging tools (Cezanne et al.).

To finalize this editorial, we encourage all readers to explore the exciting discoveries and advances in the Molecular Biology of Archaea that we have collected in this Research Topic. The archaeal community is expanding, and this Research Topic underscores how new hypotheses are continuously being proposed and ongoing research is set to reveal the unique biology of archaea. After the success of this Research Topic linked to the *Molecular Biology of Archaea 2022*, we are eagerly anticipating the upcoming Molecular Biology of Archaea meeting, which will be held in Paris this coming June.

## Author contributions

MvW: Writing – review & editing, Writing – original draft, Conceptualization. SI: Writing – review & editing, Writing – original draft, Conceptualization. MS-M: Writing – review & editing, Writing – original draft, Conceptualization. AC-O: Writing – review & editing, Writing – original draft, Conceptualization.
